# Traditional Chinese medicine may reduce the dosage of systemic glucocorticoids in required patients with acute exacerbation of chronic obstructive pulmonary disease: Study protocol for a randomized placebo-controlled trial

**DOI:** 10.1097/MD.0000000000020035

**Published:** 2020-05-01

**Authors:** Keni Zhao, Keling Chen, Qingsong Huang, Peiyang Gao, Chuantao Zhang, Hongjing Yang, Wenfan Gan, Wei Xiao, Zengtao Sun, Xiaohong Xie, Kunlan Long, Song Zhang, Jun Chen

**Affiliations:** a Department of Respiratory Medicine; b Department of Critical Care Medicine, Hospital of Chengdu University of Traditional Chinese Medicine, Chengdu, China.

**Keywords:** acute exacerbation of chronic obstructive pulmonary disease, clinical trials, dosage of systemic glucocorticoids, traditional Chinese medicine

## Abstract

**Introduction:**

Acute exacerbation of chronic obstructive pulmonary disease (AECOPD) involves a severe inflammatory response. Systemic glucocorticoids are very important for the treatment of the acute exacerbation period; however, their use causes serious adverse effects. There is still no evidence on whether traditional Chinese medicine (TCM) can be used to reduce the dosage of systemic glucocorticoids in the treatment of patients with AECOPD.

**Methods:**

In this trial, we plan to enroll 204 eligible patients with AECOPD who will be randomly assigned to receive TCM or a placebo. The effect of TCM in the treatment of patients with AECOPD will be measured by the dosage of systemic glucocorticoids (at which COPD assessment test [CAT] scores improve by 50%). Safety will also be assessed.

**Trial registration:**

ChiCTR2000029568.

## Abstract 2

Supplemental Digital Content is available in the text

## 1 Introduction

Chronic obstructive pulmonary disease (COPD) constitutes a group of diseases including small airway obstruction, emphysema, and chronic bronchitis. Chronic inflammation of the airway and lung parenchyma is the main feature of this disease, with progressive and irreversible airflow limitation.^[1]^ The main clinical manifestations are repeated cough, sputum, and dyspnea. It is usually caused by abnormal airways and alveoli caused by significant exposure to toxic particles or gases. The latest Global Initiative for Chronic Obstructive Lung Disease (GOLD) 2019 guidelines have added risk factors for COPD, such as indoor biofuels, socioeconomic status, human immunodeficiency virus infection, and genetic polymorphisms.^[2]^ Acute exacerbation of COPD (AECOPD) is defined as a sudden worsening of the main complaint (baseline dyspnea, cough, or sputum) in patients with COPD that exceeds normal day to day variations. Currently, the prevalence of COPD is on the rise. It is expected that more than 4.5 million people will die annually owing to COPD by 2030. Thus, COPD will become the third leading cause of death worldwide and a public health problem in every country.^[3,4]^

Airway inflammation is the most basic pathological change in COPD, especially in the acute exacerbation stage,^[5]^ which formed the theoretical foundation for glucocorticoid therapy. The systemic use of glucocorticoids, one of the major treatments for AECOPD,^[6]^ promotes symptom relief, shortens the course of AECOPD, reduces the length of hospital stay, and improves forced expiratory volume in 1 s (FEV1) values; however, the long-term use of steroid hormones causes muscle weakness, diabetes, osteoporosis, fluid retention, hypertension, adrenal depression,^[7]^ secondary infections (pneumocystis pneumoniae), seizures, insomnia, weight gain, anxiety, depressive symptoms, and many other significant adverse reactions.^[8]^ Each side effect is an independent risk factor for death.^[9]^ Therefore, clinicians are actively seeking safer alternative treatments that still benefit from the use of glucocorticoids.

Treatment with syndrome differentiation is one of the characteristics of the traditional Chinese medicine (TCM) theoretical system, and it is advantageous in the treatment of COPD and has achieved significant effects. “Phlegm-heat congested lung” is one of the major features of COPD.^[10]^ The TCM for clearing heat and resolving phlegm often achieves good effects in the treatment of AECOPD,^[11]^ inhibits inflammatory reactions,^[12]^ reduces airway mucus hypersecretion,^[13]^ controls lung infection, and reduces the use of antibacterial drugs.^[14]^ Based on these effects, we intend to further study the heat-clearing and phlegm-resolving effects of TCM on the reduction of the dosage of systemic glucocorticoids. In this trial, the dosage of systemic glucocorticoids will be regarded as the primary endpoint. The secondary endpoints will include: symptom scores, assessed by the COPD assessment test (CAT) scores and modified medical research council dyspnea scale scores (mMRC); exercise tolerance, measured by 6 minute walking distance (6MWD); lung function, measured by forced expiratory volume in 1 second (FEV1), the ratio of forced expiratory volume in 1 second to forced vital capacity (FEV1/FVC), and FEV1 as a percentage of predicted value (FEV1%); inflammatory indicators, include C-reactive protein (CRP) and procalcitonin (PCT); length of hospital stay; and symptoms and quality of life scores during the follow-up. The efficacy of TCM in the treatment of AECOPD will be assessed by these indicators.

## 2 Methods

### 2.1 Trial design and oversight

**Figure 1:**
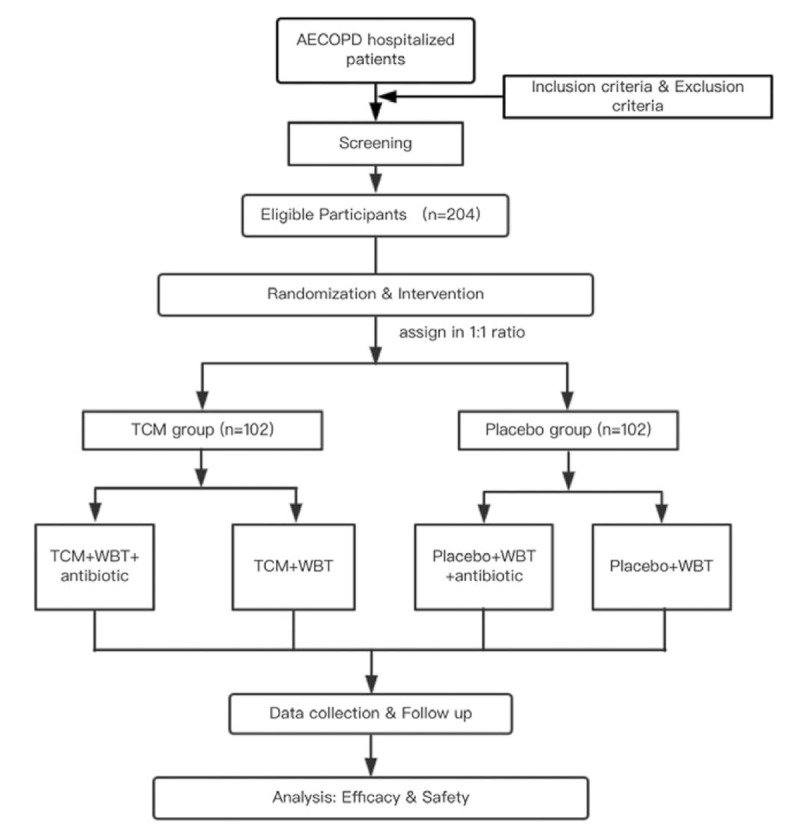
Flow chart of the study design. TCM = traditional Chinese medicine, WBT = western basic therapy.

This will be a prospective, placebo-controlled clinical trial to evaluate the efficacy of TCM in the treatment of patients with AECOPD. A total of 204 eligible patients will be enrolled according to the exclusion and inclusion criteria and randomly assigned to the TCM or placebo group at a 1:1 ratio. The antibiotic group and non-antibiotic group will be set in advance for subgroup analysis. After a 7–14 day intervention and 12 weeks of follow up, the efficacy of TCM in the treatment of patients with AECOPD will be evaluated based on the primary endpoint (the dosage of systemic glucocorticoids [at which CAT scores improve by 50%]) and secondary endpoints (symptoms scores, exercise tolerance, lung function, length of hospital stay, symptoms, and quality of life scores during the follow up). The flow chart of the study is presented in Figure 1.

This trial has been registered in the Chinese Clinical Trial Registry (ChiCTR) (ChiCTR2000029568) on February 5, 2020 and approved by the China Ethics Committee of Registering Clinical Trials (Approval No. ChiECRCT20200022). The main test center will be the Affiliated Hospital of Chengdu University of Traditional Chinese Medicine. This trial will be conducted in accordance with the Declaration of Helsinki (Edinburgh 08 version) and the Recommendations for Interventional Trials of the Standard Protocol Items (SPIRIT) initiative 2013 (see Supplemental Digital Content)^[15]^; all patients will need to provide written informed consent to proceed. If there is any amendment to the protocol, approval must be again sought from the Ethics Committee. Trial data will be uploaded to the ChiCTR within the prescribed period and published in the form of a paper in an international or domestic peer-reviewed journal.

### 2.2 Participants

This trial will begin to recruit 204 cases in June 2020. All the participants will be recruited from the inpatient department of the Respiratory Department of the Affiliated Hospital of Chengdu University of Traditional Chinese Medicine.

The western medicine diagnosis of AECOPD is similar to that of the Chinese Expert Consensus on the diagnosis and treatment of AECOPD (2017 update).^[8]^ Patients often show exacerbation of shortness of breath, often accompanied by wheezing, chest tightness, coughing, increased sputum volume, sputum color and viscosity changes, and fever. In addition, non-specific symptoms, such as tachycardia, shortness of breath, general malaise, insomnia, drowsiness, fatigue, depression, and mental disorders, may occur. Reduced exercise tolerance, fever, and chest imaging abnormalities may be clinical signs COPD.

The syndrome differentiation of “Phlegm-heat congested lung” refers to the 2012 Guidelines for Diagnosis and Treatment of COPD (2012).^[16]^ Primary symptoms include cough, wheezing, tightness in the chest, excessive phlegm production, yellow and white sticky sputum, white sticky dryness in the throat, increased expectoration, red tongue with yellow and greasy tongue coating, and a slippery or rapid pulse. Secondary symptoms include chest pain, fever, thirst for cold drinks, dry stool, and thick tongue coating. The diagnosis of phlegm-heat congested lung is based on:

1. cough or shortness of breath;
2. polychromatic, yellow, and white sticky sputum;
3. fever and thirst for cold drinks;
4. dry stool; and
5. red, yellow, or yellow greasy tongue, and a slippery or rapid pulse.

The diagnosis should meet (1) and (2) and two of (3), (4), and (5).

### 2.3 Inclusion criteria

- Patients who meet the diagnosis of phlegm-heat congested lung syndrome of AECOPD and need systemic glucocorticoid administration;
- COPD GOLD stage 3 or 4 (post-bronchodilator forced FEV1/FVC < 70% and FEV1 < 50% of predicted);
- Age from 40 to 65 years old and have symptomatic COPD (CAT scores ≥ 10), regardless of sex;
- No history of allergies to the drugs used in the experiment;
- Expected survival time ≥30 days;
- Patients and their families agree to participate and sign informed consent.

### 2.4 Exclusion criteria

- Atypical bacterial infections, such as atypical pathogens and drug-resistant bacteria;
- Complicated with a severe tumor, unstable coronary heart disease, abnormal liver and kidney function, cerebrovascular accident, and other diseases;
- Other associated active or clinically significant respiratory diseases that have a significant impact on the study, such as active pulmonary tuberculosis, lung cancer, bronchiectasis, pulmonary hypertension, pulmonary interstitial disease, and other active lung diseases;
- Severe hypertension, diabetes, peptic ulcer, and other glucocorticoids contraindications;
- Patients who require invasive mechanical ventilation;
- Pregnant, lactating, and menstrual women;
- Patients with mental disorders who are unable to actively cooperate with treatment;
- Patients with a history of allergies to related drugs.

### 2.5 Sample size

Based on the primary endpoint (the dosage of systemic glucocorticoids) to calculate sample size. According to a previous study,^[17]^ the dosage of systemic glucocorticoids during the hospitalization of patients with AECOPD with TCM is 187.6 ± 31.1 mg (methylprednisolone sodium succinate for injection) and that with western medicine treatment is 256.7 ± 32.9 mg. Using a formula, we will set α = 0.05 and β = 0.2 and hope that 80% power can be used to uncover the true difference between the treatment options. According to the formula, nc = (μ1−α / 2 + μ1−β) ^2^S^2^ (1 + 1 / k) / (μt-μc) 2 = 87. Thus, the control group requires 87 cases. According to the 1:1 ratio, a total of 174 cases are needed in the two groups. Considering a possible dropout rate of 15%, a total of 174/0.85 = 204 cases are required, with 102 cases in each group.

### 2.6 Randomization and intervention

The randomization protocol will be designed by a member of the Sichuan TCM Evidenced-Based Medicine Center. They will generate 204 random numbers will using SAS 9.2 statistical software (SAS Institute, Cary, NC). Treatment allocation will be conducted by an independent statistician according to the random numbers generated by the statistical software. Eligible patients will be randomly assigned to either the TCM group or the placebo group using sealed opaque envelops after obtaining informed consent. A special manager will record this randomization process. An individual's randomization number and group allocation can be identified by checking the envelope, which will be performed in case of a clinical emergency.

All participants will receive basic Western medical therapy advocated by the Chinese Expert Consensus on the diagnosis and treatment of AECOPD (2017 update),^[8]^ which includes:

1. Controlled oxygen therapy: Venturi mask inhales oxygen.
2. Bronchodilators: compound ipratropium bromide solution for inhalation (Laboratoire Unither, Amiens, France), 2.5 mL, inhale, three times a day;
3. Phlegm medicine: fudosteine tablets (Pharmaceutical Co., Ltd.), 0.4 mg, orally, three times a day;
4. Glucocorticoids: methylprednisolone sodium succinate for injection (Tianjing Jinyao Pharmaceutical Co., Ltd. Tianjing, China), 40 mg, administered with 0.9% sodium chloride injection (100 mL), intravenously, once a day (depending on the patient's condition, the dosage can be reduced or changed to oral administration, but the longest course of treatment cannot exceed 14 days);
5. Antibacterial drugs: according to bacterial resistance and the general course of treatment will be 5 to 7 days, although special circumstances can appropriately extend the application time of antibacterial drugs.

In addition, the TCM group will receive TCM granules (one bag at a time, three times a day) and the Placebo group will receive Placebo granules (one bag at a time, three times a day). Both groups will or will not receive antibiotic treatment according to the antibiotic treatment criteria, which are:

1. During AECOPD, aggravated difficulty breathing, increased sputum volume, and purulent sputum occur simultaneously;
2. The patient only has two of the above three symptoms, but has purulent sputum;
3. Severe acute exacerbation that requires invasive or noninvasive mechanical ventilation.

**TCM granules.** The TCM granules will be provided by the Sichuan Green Pharmaceutical Technology Development Co., Ltd. (Sichuan, China). The main components of TCM are presented in Table 1.

**Table 1:**
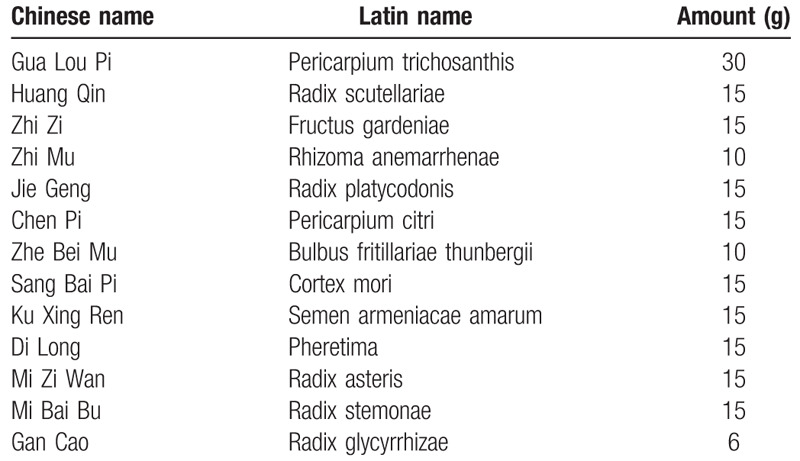
Main components of traditional Chinese medicine.

**Placebo.** The appearance and taste of the placebo, which consists of starch without any active ingredient, will be as close as possible to the TCM granules. The drug instructions for the placebo will be consistent with those for TCM granules and will be produced by the same manufacturer.

### 2.7 Length of intervention time

The entire hospital stay will be ∼7 to 14 days.

### 2.8 Discharge criteria

The theoretical discharge criteria will be^[8]^:

1. Clinicians believe that patients can adapt to home medical care;
2. Patients can use long-acting bronchodilators, such as β2 receptor agonists and anticholinergics, with or without inhaled glucocorticoids for stable inhalation therapy. Inhaled short-acting β2 receptor agonists should be administered less than once every 4 h;
3. If the patient is not bedridden, they need to be able to walk indoors;
4. Patient is able to eat and sleep and is not affected by dyspnea;
5. Patient clinically stable for 12 to 24 h;
6. Arterial blood gas analysis is stable for 12 to 24 h;
7. The patient (or family member) fully understands the proper use of the drug in the stable phase;
8. Follow-up and care plans are properly arranged (e.g., follow-up to community doctors and family medicine).

### 2.9 Follow up

Patients will be followed up by phone once a week for 12 weeks after discharge. The contents of the follow up mainly included the scores of CAT, mMRC, and 6MWD.

### 2.10 Primary outcome measure

**The dosage of systemic glucocorticoids (at which the CAT scores improve by 50%)**. Measurement methods: ([admission CAT scores − post-intervention CAT scores)/admission CAT scores) × 100%. Record glucocorticoids dosage in case report form (CRF) based on CAT scores.

### 2.11 Secondary outcome measure

**Symptoms scores**. Measured by CAT and mMRC scores. Patients will be required to complete assessments every day during hospitalization.

**Lung function**. Lung function indicators include FEV1, FEV1/FVC, and FEV1%. These will be compared before and after treatment.

**Exercise tolerance**. We will assess each patient's exercise endurance using 6MWD. Method: hospitalized patients will be asked to walk as far as possible within 6 min along flat ground once a day. For the standardized instructions of 6MWD, we will refer to the official European Respiratory Society/American Thoracic Society technical standard for field walking tests in chronic respiratory disease.^[18]^

**Inflammatory indicators**. Include CRP and PCT. We will collect the results of CRP and PCT in admissions and discharges.

**Length of hospital stay** Length of hospital stay = discharge date – admission date + 1.

**Symptoms and quality of life scores during the follow up.** Patients will be followed up by phone every week to produce the CAT, mMRC, and 6MWD scores for 12 weeks after discharge.

### 2.12 Prespecified subgroup analysis

A subgroup analysis will be performed on the use of antibiotics and no antibiotics in the TCM and placebo groups to explore the correlation between antibiotic therapy and the outcomes of the study.

### 2.13 Safety assessment

During the hospital stay, the patient will receive a daily physical examination and report to the bed physician for the recording of the symptoms and more or worse comorbidities to detect and deal with possible adverse reactions in a timely manner. Before treatment, during treatment, and on discharge, blood routine, urine routine, liver and kidney function, electrolytes, electrocardiogram (ECG), pulmonary function, blood gas analysis, and other inspections will be performed to assess whether there are adverse reactions related to treatment.

### 2.14 Study procedures

**Figure 2:**
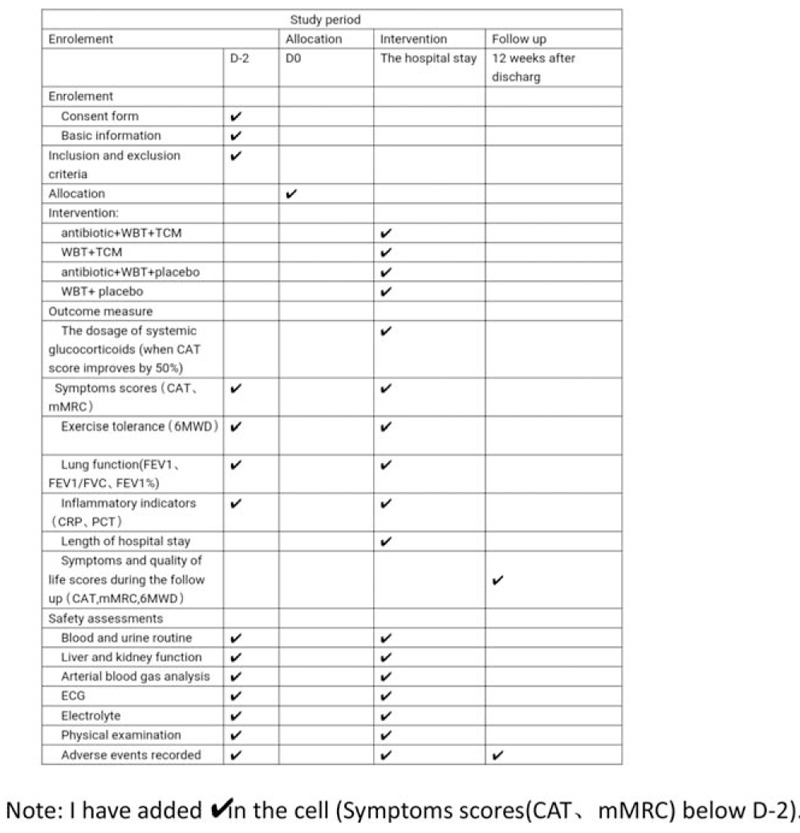
Spirit figure of enrollment, interventions, and assessments.^[15]^6MWT = 6-minute walk test, CAT = COPD assessment test, CRP = C-reactive protein, FEV1 = forced expiratory volume in 1 second, FEV1/FVC = the ratio of forced expiratory volume in 1 second to forced vital capacity, FEV1% = FEV1 as a percentage of expected value, FVC = forced vital capacity, mMRC = modified medical research council dyspnea scale scores, PCT = procalcitonin;TCM = traditional Chinese medicine, WBT = western basic therapy.

Eligible subjects will be screened 2 days before the trial. Randomized group treatment will be conducted after informed consent is obtained and baseline information is collected. The duration of the intervention will be the entire hospital stay (7–14 days). All hospitalized patients will complete the CAT, mMRC, and 6MWD scales every day. The physician should record each patient's glucocorticoid dosage every day and make corresponding adjustments according to the CAT scores. The patients will be asked about their symptoms and quality of life scores (assessed by CAT, mMRC, and 6MWD) after discharge for 12 weeks by weekly telephone follow-up. The spirit figure of enrollments, interventions, and assessments is presented in Figure 2.^[15]^

### 2.15 Quality control and data management

The study protocol has been reviewed and revised several times by the principal scientists of this protocol. To ensure that all members of the study are familiar with the study procedures and rules to ensure that the study is completed successfully, they will be required to take part in a series of training. All data will be collected by a trained and qualified investigator and recorded in CRF. To ensure the authenticity of the data, the original record will not be changed if any corrections are made once a CRF is completed.

The entire process of data entry and management will be monitored by a designated researcher. The established data will be locked by the main data managers and statistical analysts after reviewing and confirming that the data are correct. Any problems encountered after the data are locked can be corrected in the statistical analysis program after confirmation. Data audits in the middle of the trial will be performed independently by an investigator from the Department of Science Research of the Affiliated Hospital of Chengdu University of Traditional Chinese Medicine.

### 2.16 Statistical analysis

Intention-to-treat analysis and per-protocol analysis will be used. Intent-to-treat analysis means that after subjects are randomized, whether they choose to withdraw from the trial or switch to another group, their results are based on their initial grouping during data analysis. Intention-to-treat analysis will be used to analyze completed cases and dropout cases. Efficacy analysis adopts per-protocol analysis; thus, it kicks out those cases with insufficient compliance during the analysis and analyzes only the completed cases. When there is a difference between the two results, the difference will be discussed and explained.

We will use SPSS 19.0 statistical software for statistical analysis. The measured value of the data will be expressed as mean ± standard deviation. If the data follow the normal distribution and the variance is homogeneous, the *t* test will be used; however, if the data does not follow the normal distribution or the variance is uneven, the Wilcoxon rank-sum test will be sued. The Bonferroni method will be used to further compare the differences between antibiotic and non-antibiotic subgroups to explore whether antibiotics will affect the outcomes. The Chi-square test will be used for counting data and rank-sum test will be used for ranking data. All tests will be two-sided, with *P* < .05 being considered statistically significant.

## 3 Discussion

COPD has a “high morbidity and high mortality rate” worldwide, and has become a major problem for medical treatment. In particular, the acute deterioration of respiratory symptoms during the acute exacerbation period poses a serious threat to health and requires additional treatment. The 2019 GOLD guidelines state that the cause of the acute exacerbation of COPD is often respiratory infections and affirm the therapeutic role of systemic glucocorticoids in the acute phase: improving patient lung function, increasing oxygenation, and shortening hospitalization and recovery time.^[5]^ However, systemic glucocorticoids cause a variety of adverse reactions such as hyperglycemia, hypertension, osteoporosis and femoral head necrosis, and gastrointestinal bleeding and higher doses of glucocorticoids cause a higher risk of adverse reactions.^[19]^ Therefore, glucocorticoids have become a topic of concern. Whether TCM can reduce the dosage of glucocorticoids is worth research.

Currently, there are a small number of research reports on the efficacy of TCM for the reduction of the dosage of glucocorticoids in patients with AECOPD.^[20–22]^ However, these reports use small sample size and do not use the reduction in glucocorticoids dosage as the primary outcome indicator. There is no high-quality clinical evidence of randomized, blinded, and large samples. Thus, in this test, the reduction of glucocorticoids dosage will be regarded as the primary outcome to evaluate the effect of the TCM in reducing the dosage of glucocorticoids for the treatment of AECOPD.

One of the advantages of this test is that it facilitates the observation of the reduction of glucocorticoid dosage as the main outcome indicator. The dosage of glucocorticoids can be adjusted by changing the CAT score, which has guiding significance for regulating the use of clinical glucocorticoids. Secondly, this trial pre-sets a subgroup analysis based on the need for antibiotic treatment, which is in line with the clinical practice of antibiotic use. The shortcomings of this trial are that the TCM intervention time for AECOPD patients is short, which may affect the expected results, as prolonging the patient's treatment time with TCM may be beneficial to patient condition and prognosis. However, the results of this study are only applicable to a single AECOPD patient group with phlegm-heat congested lung syndrome. It will still be unknown whether patients who do not meet this syndrome can be effectively treated using the same TCM. A final disadvantage is that samples will be gathered from a single center only, which will reduce the applicability and generalizability of the results.

The purpose of this test is to observe whether the TCM for clearing heat and resolving phlegm can reduce the dosage of glucocorticoids in hospitalized patients and provide more evidence for the treatment of AECOPD by TCM.

## Acknowledgments

We are grateful to Sichuan Science and Technology Program for funding this study. We also would like to thank Editage (www.editage.cn) for English language editing.

## Author contributions

**Conceptualization:** Chuantao Zhang, Peiyang Gao.

**Investigation:** Kunlan Long, Wenfan Gan, Jun Chen.

**Supervision:** Wei Xiao, Zengtao Sun, Qingsong Huang.

**Writing – original draft:** Keni Zhao, Song Zhang.

**Writing – review & editing:** Keling Chen, Hongjing Yang, Xiaohong Xie.

Chuantao Zhang orcid: 0000-0003-1212-971X.

## Supplementary Material

Supplemental Digital Content
